# Impacto de la Pandemia por COVID-19 sobre la Atención del Infarto de Miocardio ST Elevado en el Perú

**DOI:** 10.47487/apcyccv.v1i2.22

**Published:** 2020-06-29

**Authors:** Piero Custodio-Sánchez, David Miranda, Luis Murillo

**Affiliations:** 1 Servicio de Cardiología. Unidad de Cardiología Intervencionista. Hospital Nacional Almanzor Aguinaga Asenjo Essalud, Chiclayo, Perú. Servicio de Cardiología Unidad de Cardiología Intervencionista Hospital Nacional Almanzor Aguinaga Asenjo Essalud Chiclayo Perú; 2 Servicio de Cardiología Clínica. Instituto Nacional Cardiovascular INCOR. Lima, Perú. Servicio de Cardiología Clínica Instituto Nacional Cardiovascular INCOR Lima Perú; 3 Médico residente de Cardiología. Instituto Nacional Cardiovascular INCOR. Lima, Perú. Médico residente de Cardiología Instituto Nacional Cardiovascular INCOR Lima Perú

**Keywords:** infarto de miocardio, COVID-19, SARS-CoV2., myocardial infarction, COVID-19, SARS-CoV2

## Abstract

**Objetivo:**

Comparar el número de ingresos, características clínicas y resultados terapéuticos de los pacientes atendidos por infarto de miocardio con elevación del segmento ST (IMCEST) antes y durante el estado de emergencia por COVID-19 en Perú.

**Métodos:**

Se realizó un estudio observacional analítico de cohortes retrospectivas, derivadas del PEruvian Registry of ST-segment Elevation Myocardial Infarction II (PERSTEMI II). Se comparó los pacientes atendidos por IMCEST 45 días antes y durante los 45 días iniciales del estado de emergencia por COVID-19 en Perú.

**Resultados:**

Durante los primeros 45 días del período de emergencia se encontró una disminución del 59% en el número de ingresos por IMCEST. Hubo una mayor proporción de pacientes con antecedentes de hipertensión arterial y dislipidemia. Se observó una tendencia a reducción en el acceso a terapias de reperfusión (73.5% vs 66.6%), siendo la fibrinólisis la terapia más utilizada. El motivo más frecuente de no reperfusión fue la presentación tardía >24 horas (41.7%, p=0.004). Hubo una tendencia a reducción del tiempo al primer contacto médico y del tiempo de isquemia hasta la reperfusión. Se registró menor incidencia de falla cardíaca post infarto. La mortalidad fue similar en ambos grupos (3.4% vs 2.7%).

**Conclusiones:**

La pandemia por COVID-19 en el Perú ha generado una disminución significativa en los ingresos por IMCEST, además de una tendencia a menor empleo de terapias de reperfusión. La presentación tardía de los pacientes fue la causa más frecuente de no reperfusión.

## Abstract (en)

**Purpose:**

To compare the number of admissions, clinical features and therapeutic outcomes of patients treated for acute ST-segment elevation myocardial infarction (STEMI), before and after the COVID-19 pandemic state of emergency in Peru.

**Methods:**

Observational analytical study of retrospective cohorts, derivated from the PEruvian Registry of ST-segment Elevation Myocardial Infarction II (PERSTEMI II). We compared the patients treated for STEMI, between 45 days before and during the first 45 days of the COVID-19 state of emergency in Peru.

**Results:**

During the first 45 days of the COVID-19 state of emergency, the team found a 59% decrease on the number of admissions for STEMI. There was a larger proportion of patients with high blood pressure and dyslipidemia. We noticed a decreasing trend in the access to reperfusion therapies (73% vs. 66.6%); the fibrinolysis was the most commonly used therapy. The most frequent reason for no reperfusion was the late onset >24 hours (41.7%, p=0.004). There was a trend of time reduction to first medical contact and less ischemia time to reperfusion. A lower incidence of post-infarction heart failure was registered. The mortality was similar in both groups (3.4% vs. 2.7%).

**Conclusions:**

COVID-19 pandemic in Peru has generated a significant reduction of STEMI admissions and a trend in less use of reperfusion therapies. The late onset of patients was the most common reason of not reperfusion.

## Introducción

En las dos últimas décadas, el mundo ha enfrentado tres emergencias de salud pública provocadas por la familia de Coronavirus. En el año 2002, el Síndrome respiratorio agudo severo (SARS-CoV) generó 8422 infecciones y 916 muertes. Posteriormente, en el año 2012, el Síndrome respiratorio del oriente medio (MERS-CoV) generó 1600 infecciones y 574 muertes. ^(^^1^^)^ Recientemente, en diciembre del 2019, en China, se reportó el primer caso de enfermedad por el Nuevo Coronavirus 2019 (COVID - 19) producida por el SARS-CoV2; originando hasta finales de abril del 2020, 3 millones de personas contagiadas y más de 200 000 muertes, siendo considerada en la actualidad una pandemia. ^(^^2^^,^^3^^)^ A nivel nacional, hasta finales de abril del 2020, se reportaron alrededor de 34,000 pacientes diagnosticados y más de 1000 muertes. Debido a la rápida propagación de la infección se realizó una reorganización en el sistema de salud, y se declaró el estado de emergencia con aislamiento social obligatorio en el Perú desde el 16 de marzo del 2020. ^(^^4^^)^ El resultado de este nuevo escenario ha afectado la atención de las enfermedades cardiovasculares, siendo una de ellas el infarto de miocardio con elevación del segmento ST (IMCEST). ^(^^5^

El IMCEST es una de las causas más frecuentes de enfermedad cardiovascular en la población general. ^(^^6^^)^ En el Perú se reporta una mortalidad intrahospitalaria de 10.1%,^7^^)^ la cual está influida por múltiples factores como la edad, comorbilidades y demora en la administración del tratamiento. ^(^^8^^)^ En los últimos meses, producto de la pandemia por COVID-19, se ha observado a nivel mundial una clara disminución del número de pacientes ingresados a emergencias con el diagnóstico de IMCEST y de las intervenciones coronarias percutáneas para su tratamiento. ^(^^9^^,^^10^^)^

En el Perú, no se conoce el impacto que la pandemia ha originado en la atención del IMCEST. Por ello, el objetivo del presente estudio es comparar el número de ingresos, características clínicas y resultados terapéuticos de los pacientes atendidos por IMCEST 45 días antes y durante los primeros 45 días del estado de emergencia por COVID-19 en el Perú.

## Material y Método

Realizamos un estudio observacional analítico de cohortes retrospectivas, derivadas del actual registro nacional de IMCEST: “Peruvian Registry of ST elevation myocardial infarction - II” (PERSTEMI II) del 2020; el cual, es una iniciativa para conocer la realidad del tratamiento del IMCEST en el Perú. Este viene registrando vía electrónica, todos los casos ingresados en los hospitales públicos, de las fuerzas armadas y clínicas privadas, de las principales capitales de departamento del Perú, que aceptaron participar en el estudio; inició en enero 2020 y tiene planificado terminar en diciembre 2020. Criterios de inclusión: pacientes mayores de 18 años, de ambos sexos, con síntomas isquémicos o equivalentes, con elevación persistente del segmento ST > 0.1 mV (o > 0.2 mV en V1 y V2) en 2 o más derivaciones contiguas, independientemente del tiempo de evolución, o con bloqueo completo de rama izquierda del haz de His, presumiblemente de novo. Criterios de exclusión: pacientes con síndrome coronario sin elevación del segmento ST, o embolismo coronario.

Para el presente subanálisis la primera cohorte incluyó pacientes con IMCEST diagnosticados antes de la declaratoria del estado de emergencia por el COVID 19 (dada el 16 de marzo del 2020) y se comparó con la cohorte de pacientes con IMCEST tratados luego de la declaratoria en pleno estado de emergencia. Debido a que el tiempo de cuarentena transcurrido hasta el punto de corte para este estudio fue de 45 días, se decidió comparar a los pacientes registrados en el estudio PERSTEMI II desde 45 días antes, hasta 45 días después del 16 de marzo. Por tanto, la primera cohorte comprendió los casos atendidos del 01 de febrero al 15 de marzo del 2020 y la segunda cohorte del 16 de marzo al 30 de abril del 2020. 

No se realizó la comparación con pacientes atendidos con IMCEST en los meses de marzo y abril del 2019, porque no se tienen esos datos, ya que el registro PERSTEMI II fue iniciado recién en enero del 2020.

Se evaluaron las características generales de la población (edad, sexo, lugar de atención), comparando los tiempos de isquemia, de primer contacto médico, tipo de reperfusión, porcentaje de pacientes a los que se les aplica algún tratamiento de reperfusión, porcentaje de no reperfundidos y complicaciones intrahospitalarias que incluyen mortalidad, complicación mecánica y falla cardíaca, entre ambas cohortes.

Los resultados se expresan en frecuencias y porcentajes para variables cualitativas y en medias o medianas con desviación estándar o rango intercuartil en las variables cuantitativas dependiendo de su distribución normal, la cual fue evaluada por el test de normalidad de Shapiro Wilk. 

Para el análisis estadístico se usó los test de Chi-cuadrado para variables cualitativas, T-student para variables cuantitativas de distribución normal y suma de rangos de Wilcoxon para variables cuantitativas de distribución no paramétrica. En todo caso se consideró un valor de p < 0.05 como de significancia estadística para la diferencia de dos valores. Toda la evaluación estadística se realizó con el programa STATA versión 14.0.

## Resultados

**Figura 1:**
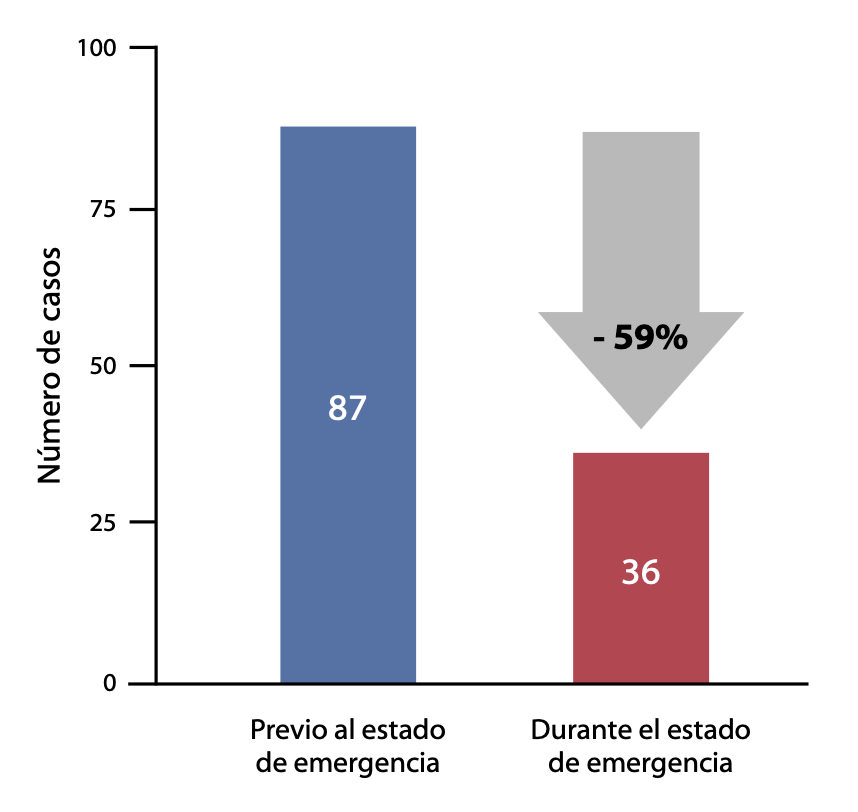
Variación en el número de casos de infarto de miocardio ST elevado, durante la pandemia por COVID -19 en el Perú.

Entre el 01 de febrero y el 30 de abril del 2020, se registraron 123 casos de IMCEST en el registro PERSTEMI II, atendidos en 9 centros de la ciudad de Lima y 6 centros del interior del país. Durante el estado de emergencia por COVID-19 (período post COVID), se evidenció una importante disminución del número de casos de IMCEST reportados en relación al período previo, lo que constituye una disminución del 59%. **(**Figura 1**)**

Dentro de las características clínicas basales no se encontraron diferencias significativas según la edad, sexo, presentación clínica y electrocardiográfica entre ambas cohortes; sin embargo, durante el estado de emergencia, encontramos una proporción significativamente mayor de pacientes con antecedente de hipertensión arterial y dislipidemia. **(**Tabla 1**)** No se reportaron casos de IMCEST en pacientes COVID - 19 positivos durante el tiempo de estudio.

**Tabla 1:** Características de los pacientes con IMCEST antes y durante el estado de emergencia por COVID - 19

	Previo al estado de emergencia (n = 87)		Durante el estado de emergencia (n = 36)		p
Edad promedio	67	(±10)	64	(±13)	0.245
Sexo masculino	73	(83.9)	33	(91.6)	0.257
Antecedentes y Factores de Riesgo					
Hipertensión arterial	39	(44.8)	25	(69.4)	0.017
Diabetes mellitus 2	30	(34.4)	8	(22.2)	0.181
Dislipidemia	39	(44.8)	25	(69.4)	0.017
Hiperuricemia	5	(3.4)	3	(8.3)	0.252
Tabaquismo	23	(26.4)	9	(25)	0.869
Enfermedad coronaria estable	6	(6.9)	4	(11.1)	0.436
Enfermedad cerebrovascular	5	(5.7)	3	(8.3)	0.597
Infarto de miocardio previo	9	(10.3)	4	(11.1)	0.9
Enfermedad renal	5	(5.7)	2	(5.5)	0.967
Falla cardíaca crónica	1	(1.2)	1	(2.7)	0.516
Ritmo por electrocardiograma					0.13
Sinusal	83	(95.4)	35	(97.2)	
Fibrilación auricular	0	(0)	1	(2.7)	
Bloqueo AV de alto grado	4	(4.6)	0	(0)	
Localización del infarto					0.73
Anterior	12	(13.9)	2	(5.5)	
Anteroseptal	19	(22.1)	6	(16.7)	
Anterolateral	4	(4.6)	3	(8.3)	
Anterior extenso	15	(17.4)	6	(16.7)	
Inferior	23	(26.4)	10	(27.8)	
Lateral	3	(3.5)	2	(5.5)	
Inferoposterior	11	(12.8)	7	(19.4)	
Presentación clínica					
Dolor anginoso	84	(96.5)	34	(94.4)	0.59
Dolor atípico	2	(2.3)	2	(5.5)	0.354
Disnea	30	(34.4)	11	(30.5)	0.674
Síncope	2	(2.3)	0	(0)	0.359
Arresto cardíaco	1	(1.1)	0	(0)	
Killip-Kimball					0.09
I	62	(71.3)	30	(83.3)	
II	23	(26.4)	4	(11.1)	
III	1	(1.1)	0	(0)	
IV	1	(1.1)	2	(5.6)	
Traslado hacia otro hospital	62	(71.3)	23	(63.9)	0.421

Se reporta medias (± desviación estándar) y frecuencias (porcentaje) para variables cuantitativas y categóricas, respectivamente.

IMCEST: infarto de miocardio con elevación del segmento ST; AV: auriculoventricular.

**Figura 2:**
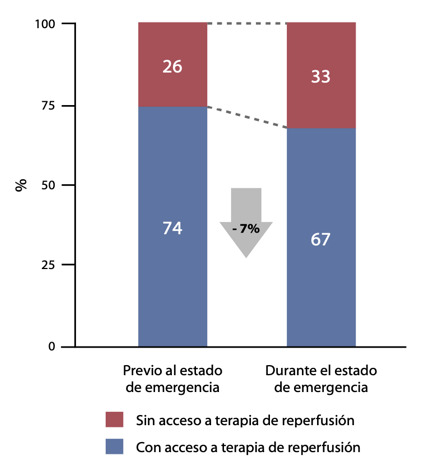
Acceso a alguna terapia de reperfusión de los pacientes con infarto de miocardio ST elevado, durante la pandemia por COVID - 19 en el Perú.

La mayoría de los pacientes recibieron alguna terapia de reperfusión (fibrinólisis o angioplastia primaria); sin embargo, se observó un menor acceso a ellas durante el estado de emergencia, **(**Figura 2**)** así como una mayor proporción de fibrinólisis y reducción de la angioplastia primaria. **(**Tabla 2**)** La presentación tardía (> 24 horas) fue el motivo más frecuente de falta de administración de alguna terapia de reperfusión durante la pandemia. **(**Tabla 2**)** Durante el estado de emergencia hubo menor tiempo al primer contacto médico, menor tiempo de isquemia hasta el uso de alguna terapia de reperfusión y un menor tiempo de isquemia hasta la intervención coronaria percutánea primaria, aunque esto no fue estadísticamente significativo. **(**Tabla 3**)** La terapia hospitalaria recibida en ambas etapas no presentó diferencias importantes. La mortalidad intrahospitalaria fue similar en ambas cohortes y se encontró menor proporción de falla cardíaca post infarto durante el estado de emergencia. **(**Tabla 4**)**

**Tabla 2:** Terapias de reperfusión administradas a los pacientes del estudio

	Previo al estado de emergencia (n = 87)		Durante el estado de emergencia (n = 36)		p
Alguna terapia de reperfusión	64	(73.5)	24	(66.7)	0.441
ICP primaria	36	(41.4)	11	(30.6)	0.261
FIbrinólisis	28	(32.1)	13	(36.1)	0.612
Éxito de la fibrinólisis	17	(60.7)	10	(76.9)	0.308
ICP de rescate	7	(8.0)	2	(5.6)	0.629
Estrategia farmacoinvasiva	13	(14.9)	7	(19.4)	0.538
Total de coronariografías e ICP	56	(64.4)	20	(55.5)	0.360
Flujo TIMI 3 final post ICP	43	(76.8)	18	(90)	0.670
Enfermedad multivaso	27	(48.2)	13	(65.0)	0.630
ICP de la ANRI	20	(74.1)	5	(45.4)	0.092
Ninguna terapia de reperfusión	23	(26.4)	12	(33.3)	0.441
Contraindicación a fibrinólisis	0	(0)	0	(0)	
Negación del paciente	1	(4.3)	1	(8.3)	0.679
Falta de fibrinolíticos	5	(21.7)	0	(0)	0.062
No disponibilidad de ICP	6	(26.1)	0	(0)	0.030
Presentación tardía > 12 horas	2	(8.7)	2	(16.7)	0.720
Presentación tardía > 24 horas	0	(0)	5	(41.7)	0.004
Presentación tardía > 72 horas	6	(26.1)	1	(8.3)	0.102
Otros	5	(21.7)	2	(16.7)	0.339
Desconocido	2	(8.7)	0	(0)	

Se reporta frecuencia (porcentaje) para cada variable.

IMCEST: infarto de miocardio con elevación del segmento ST; ICP: intervención coronaria percutánea;

ANRI: arteria no responsable del infarto.

**Tabla 3:** Componentes del tiempo de isquemia en los pacientes de estudio

	Previo al estado de emergencia (n = 87)		Durante el estado de emergencia (n = 36)		p
Tiempo hasta el primer contacto médico (minutos)	120	(60 - 240)	90	(45 - 180)	0.281
Tiempo de isquemia hasta la aplicación de alguna terapia de reperfusión (minutos)	330	(240 - 1260)	237	(150 - 480)	0.086
Tiempo de isquemia hasta ICP primaria (minutos)	690	(330 - 1500)	480	(225 - 1200)	0.243
Tiempo puerta - balón (minutos)	90	(50 - 180)	60	(45 - 100)	0.634

Se reporta mediana (rango intercuartil) para cada variable.

ICP: intervención coronaria percutánea

**Tabla 4:** Terapias utilizadas y eventos intrahospitalarios en los pacientes de estudio

	Previo al estado de emergencia (n = 87)		Durante el estado de emergencia (n = 36)		p
Terapia hospitalaria					
Inotrópicos	11	(12.6)	2	(5.5)	0.245
Vasopresores	10	(11.5)	2	(5.5)	0.313
Balón de contrapulsación intraaórtico	4	(4.6)	0	(0)	0.191
Ventilación mecánica	11	(12.6)	2	(5.5)	0.245
Eventos intrahospitalarios					
Muerte cardiovascular	3	(3.4)	1	(2.7)	0.849
Muerte no cardiovascular	2	(2.3)	0	(0)	0.359
Complicaciones mecánicas	2	(2.3)	1	(2.7)	0.876
Shock cardiogénico	7	(8.1)	3	(8.3)	0.958
Falla cardíaca	21	(24.1)	3	(8.3)	0.048
Stroke	1	(1.1)	0	(0)	0.518

Se reporta frecuencia (porcentaje) para cada variable.

## Discusión

El presente subanálisis del registro PERSTEMI II muestra una reducción del 59% en los ingresos por IMCEST durante el estado de emergencia por COVID-19 en el Perú. Este hallazgo es llamativo, ya que existe evidencia de factores que pueden incrementar la incidencia de infarto de miocardio durante infecciones respiratorias agudas. Kwong *et al*. ^(^^11^^)^ reportaron la asociación significativa entre el infarto de miocardio y la infección por influenza durante los primeros 7 días de la misma. Igualmente, durante la infección por coronavirus responsable del SARS aparecido en China el año 2002, se reportaron casos de infarto de miocardio en relación a la inflamación sistémica aguda que generaba el virus. ^(^^12^^,^^13^

Asimismo, existen factores psicosociales que pueden desarrollarse durante el período de cuarentena, como el estrés agudo relacionado por temor al contagio de la infección o los trastornos de ansiedad y depresión propios del aislamiento, los cuales pueden comportarse como gatillos de infarto de miocardio. ^(^^14^^,^^15^^)^ Adicionalmente, la cuarentena puede condicionar la falta de adherencia de los pacientes a su terapia habitual ante la dificultad para conseguir medicación por restricciones de movimiento o el temor al contagio de la infección al acudir a hospitales, lo que lleva a la pérdida de control de los factores de riesgo tradicionales modificables como la hipertensión arterial, diabetes y dislipidemia.

A pesar de la evidencia precedente, lo que se aprecia en nuestro registro y en los realizados en otros países, es una disminución importante en el número de pacientes atendidos por IMCEST. El trabajo publicado por Rodríguez Leor *et al*. ^(^^5^^)^ describe una reducción del 40% en el intervencionismo coronario percutáneo por IMCEST en 81 centros españoles que respondieron a una encuesta sobre la actividad asistencial en sus instituciones. La misma tendencia ha sido reportada por García *et al*. ^(^^10^^)^ quienes describen una reducción de 38% en las activaciones de los laboratorios de cateterismo cardíaco por IMCEST en 9 centros de alto volumen en los Estados Unidos. Reducciones menores se describen en Italia donde De Filippo *et al*. ^(^^16^^)^ reportaron una caída de 22% en las admisiones por IMCEST en 15 hospitales de la región de Lombardía. Aunque los estudios descritos se han realizado en algunas de las zonas más afectadas por la pandemia del COVID-19 también se reproduce la tendencia en zonas de menor afectación como es el caso de Austria donde Metzler *et al*. ^(^^17^^)^ encuentran una reducción de 39.4% en las admisiones por síndrome coronario agudo y 26% por IMCEST.

Se han planteado múltiples hipótesis respecto a las causas de la reducción del volumen de casos atendidos de IMCEST. Una primera posibilidad a mencionar es el temor que tiene el paciente a acudir a un centro hospitalario donde podría contagiarse con la infección. Este aspecto ha sido evidenciado por la encuesta realizada por el Colegio Americano de Médicos de Emergencias donde el 80% de los encuestados manifiesta su temor al contagio al acudir a una sala de emergencia y 29% refiere que evitará cualquier consulta médica durante la pandemia. ^(^^18^^)^ Al no acudir en busca de atención médica es posible que se presente un incremento de las complicaciones del IMCEST no reperfundido incluida la muerte súbita. Baldi *et al*^19^^)^ reportaron un aumento de 23% en la incidencia de arresto cardíaco extra-hospitalario no asociado a COVID-19 en 4 provincias de Lombardía, Italia. También se ha planteado un sub-diagnóstico como causa de la reducción de ingresos por IMCEST, ya que algunos síntomas del infarto de miocardio podrían solaparse con los del COVID-19 como reportan Yousefzai *et al*. ^20^

Nuestro registro muestra una tendencia a que los pacientes reciban menos terapias de reperfusión; con incremento en el uso de fibrinólisis y reducción de la angioplastia primaria lo que podría corresponder a cambios en protocolos de manejo y gestión de salas de hemodinámica durante la pandemia como ha sido reportado por Rodríguez-Leor *et al*. ^(^^5^^)^ No hubo variación estadísticamente significativa en los tiempos evaluados (primer contacto médico, isquemia a reperfusión, puerta-balón), con una tendencia a la reducción del tiempo al primer contacto médico, a diferencia de la serie de casos reportada por Tam *et al*. ^(^^9^^)^ en un hospital de Hong Kong donde se evidenció la prolongación de los tiempos mencionados. Este hallazgo podría explicarse por la reducción del tiempo de transporte de nuestros pacientes, al estar restringida la circulación vehicular durante el período de estado de emergencia salvo para emergencias médicas. Asimismo la reducción del tiempo de isquemia a reperfusión podría justificar la menor incidencia de falla cardíaca post infarto a pesar de la reducción en las terapias de reperfusión.

A pesar de no encontrar diferencias significativas en los tiempos promedio de atención ni en mortalidad hay un aspecto importante a destacar que es el incremento significativo de la proporción de pacientes con presentación tardía (>24 horas) como motivo de no reperfusión; es posible que el temor de contagio al acudir al hospital explique este hallazgo.

Como limitaciones de este subanálisis podemos señalar que si bien el registro PERSTEMI II del 2020 es de alcance nacional, los datos evaluados corresponden principalmente a centros pertenecientes al sistema público de salud ubicados en la ciudad de Lima y el Callao, por lo que sus conclusiones no necesariamente reflejan la situación de los ingresos por IMCEST en todo el territorio nacional. Se realizó un análisis retrospectivo, y no fue posible realizar la comparación con los meses correspondientes del año 2019, al no contar con dicha información.

## Conclusiones

El presente subanálisis del registro PERSTEMI II muestra una reducción del 59% en los ingresos por IMCEST durante los primeros 45 días del estado de emergencia por COVID-19 en el Perú respecto a los 45 días previos a éste. No hubo variación significativa en los tiempos de primer contacto médico, isquemia a reperfusión y puerta balón; sin embargo se evidenció tendencia a menor empleo de terapias de reperfusión y se incrementó la presentación tardía de los pacientes (>24 horas) como causa de no reperfusión.

Esta reducción de las atenciones por IMCEST, podría tener como consecuencia el incremento de morbimortalidad, lo que obliga a las autoridades sanitarias a informar a la población en general sobre los síntomas y signos de alarma que deben motivar la consulta hospitalaria de emergencia.

## Financiación:

Ninguna
